# Rapid optimization of spore production from *Bacillus amyloliquefaciens* in submerged cultures based on dipicolinic acid fluorimetry assay

**DOI:** 10.1186/s13568-018-0555-x

**Published:** 2018-02-16

**Authors:** Hang Ren, Ya-ting Su, Xiao-hua Guo

**Affiliations:** Provincial Key Laboratory for Protection and Application of Special Plants in Wuling Area of China, College of Life Science, South-Central University for Nationalities, No. 182, Minyuan Road, Hongshan District, Wuhan, 430074 Hubei China

**Keywords:** *Bacillus amyloliquefaciens*, Spore yields, Response surface methodology, Dipicolinic acid, Fluorescence intensity

## Abstract

Some optimization techniques have been widely applied for spore fermentation based on the plate counting. This study optimized the culture medium for the spore production of *Bacillus amyloliquefaciens* BS-20 and investigated the feasibility of using a dipicolonic acid (DPA) fluorimetry assay as a simpler alternative to plate counting for evaluating spore yields. Through the single-factor experiment, the metal ions and agro-industrial raw materials that significantly enhanced spore production were determined. After conducting a response surface methodology (RSM) analysis of several metal ions, the combined use of optimum concentrations of Mn^2+^, Fe^2+^, and Ca^2+^ in culture media produced a 3.4-fold increase in spore yields. Subsequently, supplementing soybean meal and corn meal with optimum concentrations determined by another RSM analysis produced an 8.8-fold increase. The final spore concentration from a culture medium incorporating optimum concentrations of the metal ions and raw materials mentioned above was verified to reach (8.05 ± 0.70) × 10^9^ CFU/mL by both DPA fluorimetry and plate counting. The results suggest that the use of DPA fluorescence intensity as an alternative value to colony counting provides a general method for assessing spore yields with less work and shorter time.

## Introduction

*Bacillus* species are aerobic or facultative anaerobic, sporulating, rod-shaped bacteria (Driks 2002). They can form protective endospores that allow them to tolerate harsh environmental stress, such as heat, radiation, desiccation, freezing and chemical disinfectants (Setlow 2006). The *Bacillus* spores can survive through the digestive process and germinate within the digestive tract (Casula and Cutting 2002). As a common source of probiotic supplements, *Bacillus* is often used in animal feeds, human dietary supplements and even in medicines (Cutting 2011).

Endospores of *Bacillus* are formed after the exponential phase of vegetative cell growth as a result of nutrient depletion and cell accumulation (Driks 2002). In the commercialization of *Bacillus*-based bio-products, high spore yields in bioreaction with less cost are preferred in industrial exploitation (Chen et al. 2010; Khardziani et al. 2017; Lalloo et al. 2009; Posada-Uribe et al. 2015). The regulation of sporulation parameters in fermentation was often carefully considered for enhanced spore production (Monteiro et al. 2005; Rao et al. 2007).

In early reports, the optimization of culture media and culture conditions was largely studied for higher spore yields for particular *Bacillus* strains, since each strain differed from different nutrient requirements and culture conditions (Chen et al. 2010; Khardziani et al. 2017; Posada-Uribe et al. 2015; Setlow 2006; Shi and Zhu 2007). In whichever reports, the spore concentrations were all quantified by plate counting assay, which were generally time-consuming and quite tedious (Hazan et al. 2012). Some alternative techniques on specific detection of spores were developed (Ai et al. 2009; He et al. 2003; Hindle and Hall 1999; Pellegrino et al. 2002), one of which was fluorimetry assay based on dipicolinic acid (DPA). DPA is a universal and specific component of bacterial spores and the limit of detection (LOD) on spores based on the DPA fluorimetry assay has reached 1000 spores/mL in the report of Pellegrino et al. (2002). The fluorimetry assay is comparatively simple, time-saving, and especially suitable for the simultaneous detection with many analytes (Pellegrino et al. 2002). However, till now, no reports were focused on spore production based on the specific DPA fluorimetry assay during the optimization procedure.

Using a statistical experiment design, this study determined the optimum concentrations of metal ions and raw materials to enhance the spore production of the potentially probiotic strain of *Bacillus amyloliquefaciens* BS-20 under submerged fermentation. In addition, the feasibility of using a DPA fluorimetry assay to quantify spore yields as the response variable in the optimization procedures was investigated.

## Materials and methods

### Bacterial strains and culture condition

*Bacillus amyloliquefaciens* BS-20, previously screened as probiotics from *Bacillus* species, was used as the starter cultures in submerged fermentation, and the isolate was deposited in the China Center for Type Culture Collection (CCTCC) as No. M 2017587. The strain was maintained at − 80 °C in 20% sterile glycerol until needed. The medium was initially developed for the maximum cell growth based on Luria–Bertani (LB) broth and was composed of: glucose 8 g/L, beef extract 7.2 g/L, NaCl 10 g/L, pH 7.0. The medium was autoclaved at 121 °C for 15 min and then used as the initial broth for the strain’s growth and spore production. The culture was kept in a 250 mL Erlenmeyer flask containing 50 mL of broth. After inoculating 2% freshly prepared culture with an initial cell concentration of approximately 2 × 10^7^ cells/mL, spore fermentation began. All experiments were carried out in a rotating shaker at 200 rpm and 37 °C. The samples were cultured for 48 h and then harvested.

### Spore detection

The spore concentration was quantified using the DPA marker in spores based on a technique described in previous reports with some modifications (Hindle and Hall 1999; Pellegrino et al. 2002). The principle of detection is that in the presence of the chelating agent cyclohexanediamine tetraacetic acid (CyDTA), DPA and the lanthanide metal europium produce a specific fluorescence excited by ultraviolet light, the intensity of which is in proportion to the concentration of DPA.

Specifically, the spores from the fermentation broth were harvested for analysis by centrifugation (2500×*g* for 10 min) and washed twice, and suspended in sterile Tris–HCl (50 mM, pH 8.0). The spore suspensions were then treated at 121 °C for 5–10 min for the full release of DPA into the buffer based on based on an earlier study (data not shown). The DPA-containing supernatants were collected after being centrifuged at 2500×*g* for 10 min. With a certain dilution, the supernatants were assayed for fluorescence intensity by mixing EuCl_3_ (2 mM) and CyDTA (2 mM) with the proportion 1:4.5:4.5 by a vortex oscillator. Meanwhile, in the fluorescent complex, DPA supernatants were replaced by isometric Tris–HCl buffer to serve as a blank control. A Hitachi F-7000 spectrofluorophotometer (Hitachi Ltd., Tokyo, Japan) was used to detect the fluorescence intensity at the excitation wavelength of 272 nm and emission wavelength of 619 nm. The scanning speed was pre-set to 3000 nm/min, the slit to 5 nm/10 nm, the photo-multiplier tube (PMT) voltage to 700 V, and the responding time to 0.08 s. In order to keep the accuracy of measurement, the DPA samples were serially diluted to make the light output in arbitrary units on a scale from 0 to 1000.

A traditional plate-counting assay was performed to verify the reliability of the DPA fluorimetry assay on spore detection. Spores were counted by heating dilutions of the culture at 80 °C for 15 min to kill vegetative cells before they were plated onto an LB agar medium. The colonies were counted after cultivation at 37 °C for 24 h, and the final results were expressed as colony-forming units per mL (CFU/mL).

The spore suspensions with the initial optical density about OD_600 nm_ of 1.0 were twofold serially diluted and the ensuing DPA fluorescence intensity was detected. The concentration of spore suspensions was detected by plate counting and DPA fluorimetry assay, respectively. The linear correlation between spore concentrations (CFU/mL) and the fluorescence intensity (AU) was built.

### Screening of significant metal ions for spore production

Six metal ions, Mn^2+^, Fe^3+^, Fe^2+^, Ca^2+^, Mg^2+^ and Zn^2+^, were identified as key factors in improving spore production based on previous reports (Granger et al. 2011; Kihm et al. 1988; Kolodziej and Slepecky 1964; Oh and Freese 1976). A single-factor experiment was carried out by adding metal ions into the autoclaved basal medium, which contained 8 g/L of glucose and 7.2 g/L of beef extract. The metal ions were filter-sterilized and added into the basal medium to reach the final concentrations listed in Table 1. The initial broth (glucose 8 g/L, beef extract 7.2 g/L, NaCl 10 g/L) served as a control. Both media were inoculated with *B. amyloliquefaciens* BS-20 and cultivated for 48 h. The harvested culture was immediately treated and quantified by DPA fluorimetry assay. The results were expressed as the means of fluorescence intensity and their standard deviations (SDs) based on three replicates. The data were analyzed by Student’s t test in the JMP11.0 software (SAS Institute Inc., USA). *P* values less than 0.05 were regarded as a significant difference. The metal ions that showed a significant positive influence on spore production were selected for optimization by a central composite design (CCD) experiment and response surface methodology (RSM) analysis.

**Table 1:** Effects of metal ions with different concentrations on the spore yields of *B. amyloliquefaciens* BS-20 detected by the fluorometric assay

Concentration (mM)	Fluorescence intensity (AU)					
	Fe^3+^	Fe^2+^	Mn^2+^	Mg^2+^	Ca^2+^	Zn^2+^
0.0	996.7 ± 48.5	996.7 ± 48.5^a^	996.7 ± 48.5^a^	996.7 ± 48.5^a^	996.7 ± 48.5^a^	996.7 ± 48.5^a^
0.5	–	–	1080.0 ± 112.8^a^	–	–	–
1.0	1073.0 ± 26.5	1205.0 ± 108.9^a^	1661.5 ± 102.5^b^	1184.5 ± 71.4^b^	1067.0 ± 98.9^ab^	285.0 ± 18.4^b^
2.0	1019.0 ± 34.0	1527.0 ± 145.7^b^	1451.0 ± 75.0^ab^	1186.0 ± 19.8^b^	1526.5 ± 64.3^c^	226.0 ± 29.7^bc^
3.0	1104.0 ± 68.5	1624.5 ± 163.3^b^	1231.5 ± 79.9^ab^	1539.5 ± 77.1^c^	1451.0 ± 93.3^c^	175.0 ± 15.6^c^
4.0	1098.0 ± 55.5	1182.5 ± 120.9^a^	–	1097.5 ± 3.5^bc^	1219.0 ± 4.2^b^	65.0 ± 9.9^d^
5.0	1012.0 ± 29.5	1136.0 ± 90.5^a^	–	1068.5 ± 62.9^bc^	1203.5 ± 34.6^b^	35.0 ± 4.2^d^

Mean values in the same column with different letters (a, b, c, d) are significantly different (*P* < 0.05). The final results are expressed as the mean ± standard deviation (n = 3) of 100-fold diluted spore samples

### Ion optimization by central composite design

A CCD and RSM analysis were employed to investigate the optimal combination of the metal ions. The RSM was applied through the statistical software JMP 11 (SAS Institute Inc., USA). The optimal concentrations of the key metal ions identified by the single-factor experiment were determined by studying each factor at five different levels: −a, −, 0, +, A (Table 2), which represented low star point, low central point, center point, high central point and high star point, respectively. For each factor, the central coded value was considered as zero, and the concentrations at the zero points were the values that significantly contributed to the highest fluorescence intensity in the single-factor experiment. The axial value was set as 1.483. The CCD was undertaken in 27 runs including 3 replicates of central point. The fluorescence intensity produced by the harvested spores was used as the response value for experimental analyses. The quadratic models for RSM were used to predict the co-effect of metal ions. The optimum concentration points for maximum spore production was determined based on the quadratic Eq. (1).

1 $$y = \beta_{0} + \sum\limits_{i = 1}^{k} {\beta_{i} {\text{x}}_{i} } + \sum\limits_{i = 1}^{k} {\beta_{ii} {\text{x}}_{i}^{2} } + \sum\limits_{i < j}^{k} {\beta_{ij} {\text{x}}_{i} {\text{x}}_{j} }$$

**Table 2:** Central composite design for metal ion factors associated with spore density by the fluorometric assay

Run	Models	Metal ions concentration (mM)				Fluorescence intensity (AU)	
		Mn^2+^ (x_1_)	Fe^2+^ (x_2_)	Ca^2+^ (x_3_)	Mg^2+^ (x_4_)	Experimental	Predicted
1	0000	1	3	2	3	299.2 ± 44.7	299.1
2	0a00	1	1.52	2	3	292.1 ± 38.3	290.3
3	−−++	0.5	2	3	4	280.8 ± 21.2	278.4
4	+−++	1.5	2	3	4	284.4 ± 38.2	286.3
5	++++	1.5	4	3	4	282.3 ± 30.0	282.2
6	−+++	0.5	4	3	4	268.2 ± 24.3	268.0
7	−−−+	0.5	2	1	4	263.4 ± 20.2	265.1
8	++−−	1.5	4	1	2	280.8 ± 28.6	282.0
9	++−+	1.5	4	1	4	281.5 ± 30.4	276.7
10	000A	1	3	2	4.48	288.5 ± 27.6	290.4
11	−−+−	0.5	2	3	2	280.5 ± 23.3	284.4
12	a000	0.26	3	2	3	278.8 ± 20.1	278.8
13	+−−+	1.5	2	1	4	266.4 ± 17.4	265.8
14	0000	1	3	2	3	302.9 ± 33.1	299.1
15	−+−−	0.5	4	1	2	287.2 ± 25.7	284.4
16	+−+−	1.5	2	3	2	284.5 ± 24.0	283.1
17	+−−−	1.5	2	1	2	271.0 ± 18.4	270.4
18	+++−	1.5	4	3	2	282.2 ± 24.6	279.6
19	00a0	1	3	0.52	3	273.9 ± 23.6	277.6
20	0A00	1	4.48	2	3	285.5 ± 23.3	291.2
21	A000	1.74	3	2	3	279.1 ± 26.0	282.9
22	−−−−	0.5	2	1	2	280.1 ± 4.7	279.0
23	−++−	0.5	4	3	2	275.2 ± 17.4	274.6
24	00A0	1	3	3.48	3	285.4 ± 19.5	285.6
25	−+−+	0.5	4	1	4	269.6 ± 15.3	269.8
26	000a	1	3	2	1.52	296.9 ± 24.5	298.8
27	0000	1	3	2	3	302.2 ± 26.6	299.1

The experimental results are the means of two replicates of 1000-fold diluted spore samples. The symbols in the model column mean each factor at five different levels (−a, −, 0, +, A). The variables at a central coded value are considered at zero

### Selection of significant raw materials for spore enhancement

Different agro-industrial materials including corn meal, soybean meal, wheat bran and molasses (about 48% sugars) were bought locally. In a similar way as the metal ions were optimized, the raw materials were added to the medium containing optimized ions and further analyzed by another single factor experiment with the same design as that described in the previous section (Table 3). The basal medium that contained these raw materials were autoclaved at 121 °C for 15 min, and the optimized ions were then added after filter-sterilization. The basal medium that only contained the optimized metal ions was used as a control.

**Table 3:** Effects of different raw materials on the spore yields of *B. amyloliquefaciens* BS-20 detected by the fluorometric assay

Concentration (g/L)	Fluorescence intensity (AU)			
	Corn meal	Soybean meal	Wheat bran	Molasses
0	304.5 ± 19.1^a^	304.5 ± 19.1^a^	304.5 ± 19.1	304.5 ± 19.1
5	392.0 ± 35.4^b^	395.0 ± 29.7^b^	305.0 ± 15.6	316.0 ± 12.7
10	525.0 ± 41.0^c^	505.0 ± 17.0^c^	325.0 ± 19.8	308.0 ± 15.6
15	483.0 ± 25.5^c^	468.0 ± 25.5^c^	309.0 ± 18.4	298.0 ± 8.5

Mean values in the same column with different letters (a, b, c) are significantly different (*P* < 0.05). The final results are expressed as the mean ± standard deviation (n = 3) of 1000-fold diluted spore samples

### Raw materials optimization by central composite design

The single-factor experiment identified the key raw materials to include for enhancing spore yields. To determine the optimum combination of raw materials, similar procedures to those used for optimizing the ions by a CCD and an RSM analysis were carried out. Similar procedures as described in ion optimization by CCD and RSM were carried out.

### Validation of the optimization procedures

After optimizing the ions and raw materials, verification experiments were carried out to check whether the spore concentrations quantified by the fluorimetry and plate counting assay were consistent. The initial broth was used as a control. The results were expressed as the means of fluorescence intensity or CFU/mL and their standard deviations (SD) based on three replicated experiments.

## Results

### DPA fluorimetry assay for quantifying the spore concentration

**Fig. 1:**
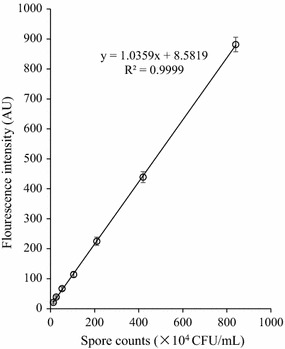
Calibration curves of spore counts of *B. amyloliquefaciens* BS-20 and their fluorescence intensity. The spores of *B. amyloliquefaciens* BS-20 with 1.34 × 10^8^ CFU/mL were twofold serially diluted and treated for the detection of fluorescence intensity

Figure 1 shows the good linear correlation between the spore concentrations varying from 8 × 10^3^ to 8 × 10^6^ CFU/mL, and corresponding DPA fluorescence intensity (coefficient R^2^ = 0.9999). The limit of detection (LOD) reached 8000 spores/mL. As a result, the fluorimetry assay was used in the following optimization procedures for spore production.

### Effect of metal ions on spore yields

Of the six metal ions, four ions including Mn^2+^, Fe^2+^, Ca^2+^, and Mg^2+^ showed significant positive influence on the enhancement of sporulation compared with the control (*P* < 0.05) (Table 1). The optimum concentrations of metal ions were 1.0 mM of Mn^2+^, 3.0 mM of Fe^2+^, 2.0 mM of Ca^2+^, and 3.0 mM of Mg^2+^, respectively.

### Ion optimization by a response surface methodology analysis

The significant metal ions chosen from the one-factor experiment, Mn^2+^ (*x*_1_), Fe^2+^ (*x*_2_), Ca^2+^ (*x*_3_) and Mg^2+^ (*x*_4_) were included in the CCD for the determination of their optimum concentrations, and the results are listed in Table 2. As observed from Table 2, the response variable was analyzed through RSM and a standard analysis of variance (ANOVA) (Table 5). The dataset could be fitted with a regression quadratic equation as described in Eq. (2).

2 $$\begin{aligned} {\text{Y }} &= { 198}.0 8 { } + { 38}. 8 8x_{ 1} + { 28}. 3x_{ 2} + { 36}. 5 7x_{ 3} + { 1}. 5x_{ 4} \\ & \quad + { 3}. 1 4x_{ 1} x_{ 2} + { 3}. 6 6x_{ 1} x_{ 3} - { 3}. 7 8x_{ 2} x_{ 3} + { 4}. 6 4x_{ 1} x_{ 4} - \, 0. 1 7x_{ 2} x_{ 4} \\ & \quad + { 1}. 9 7x_{ 3} x_{ 4} - { 33}. 3 7x_{ 1}^{ 2} - { 3}. 8 4x_{ 2}^{ 2} - { 8}.0 2x_{ 3}^{ 2} - { 2}.0 6x_{ 4}^{ 2} \hfill \\ \end{aligned}$$

The model showed the optimization was successful in improving spore production since the coefficient of determination, *R*^2^, and adjusted determination coefficient Adj. *R*^2^ were 0.94 and 0.87, respectively. The value of “*P* > *F*” was less than 0.05, indicating that the model was significant. The terms $x_{1}^{2}$, $x_{3}^{2}$, *x*_3_, *x*_2_*x*_3_, *x*_2_, $x_{2}^{2}$, *x*_1_ and *x*_1_*x*_4_ (arranged by ascending *P* values) were found to be significant (*P* < 0.05). For the other model terms associated with the variable Mg^2+^ (i.e. $x_{4}^{2}$, *x*_4_, *x*_2_*x*_4_), the *P* values were 0.1026, 0.8547 and 0.8588, respectively. Therefore, Mg^2+^ (*x*_4_) might play less roles in interacting with other metal ions in sporulation. A complementary experiment was undertaken to test the effect of the ion-optimized medium in the presence or absence of Mg^2+^. No significant difference in spore yields was observed (data were not shown). In order to lower the number of variable in final medium, Mg^2+^ was not considered in the further study.

**Fig. 2:**
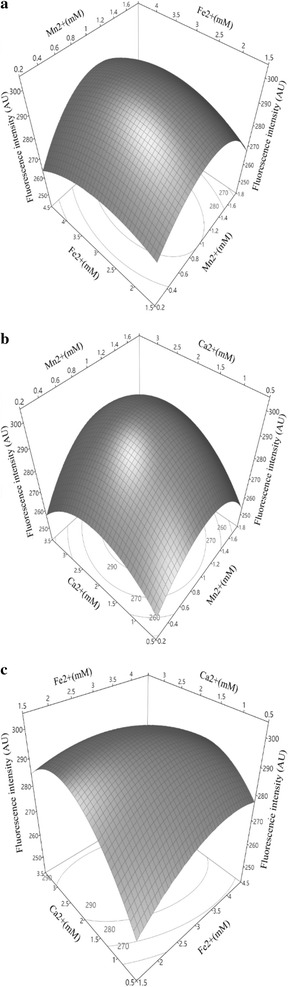
Response surface plots for spore production caused by metal ions. The interaction between **a** Mn^2+^ and Fe^2+^, **b** Fe^2+^ and Ca^2+^, **c** Mn^2+^ and Ca^2+^, respectively

Response surface plots were drawn to study the interactive effects of metal ions on sporulation and to determine their optimum concentrations for maximum possible spore yields (Fig. 2a–c). The response surface and contour plots indicated that the interactions between the independent variables Mn^2+^ (*x*_1_), Fe^2+^ (*x*_2_) and Ca^2+^ (*x*_3_) were significant. All three response surface plots had a convex surface with a downward opening shown in Fig. 2. Therefore, the response surface maximal point (300.02 AU) was obtained when the optimal significant variables were at the following levels: Mn^2+^ (*x*_1_) = 1.0 mM, Fe^2+^ (*x*_2_) = 3.0 mM, Ca^2+^ (*x*_3_) = 2.1 mM.

### Effect of raw materials on spore yields

On the basal medium containing the optimized concentration of metal ions, the effects of four main raw materials on the spore yields conducted in a one-factor experiment are presented in Table 3. Corn meal and soybean meal positively influenced spore production (*P* < 0.05). However, no significant effect was found from wheat bran and molasses (*P* > 0.05). The co-effect of corn meal and soybean meal was further studied in a CCD and RSM analysis over 11 runs, including 3 replicates of central point.

### Raw materials optimization by response surface methodology

The design and result of the CCD from the corn and soybean meal variables are presented in Table 4, and the RSM analysis and ANOVA are presented in Table 5. The quadratic regression is described in Eq. (3).

3 $${\text{Y }} = \, - 5 5 8 8. 8 4 { } + { 559}. 1x_{ 5} + { 812}. 4 8x_{ 6} - { 6}. 7 3x_{5} x_{6} - 2 7. 4 8x_{ 5}^{ 2} - { 39}. 4 3x_{ 6}^{ 2}$$

**Table 4:** Central composite design for soybean meal and corn meal associated with spore density by the fluorometric assay

Run	Models	Concentration (g/L)		Fluorescence intensity (AU)	
		Corn meal (x_5_)	Soybean meal (x_6_)	Experimental	Predicted
1	A0	11.4	10	626.5 ± 47.4	628.5
2	00	10	10	750.4 ± 55.4	763.2
3	+−	11	9	672.4 ± 38.4	688.7
4	0A	10	11.4	615.9 ± 26.2	625.2
5	00	10	10	765.6 ± 55.5	763.2
6	00	10	10	772.9 ± 59.9	763.2
7	−+	9	11	711.3 ± 54.1	717.4
8	0a	10	8.6	779.0 ± 58.6	746.7
9	a0	8.6	10	815.2 ± 55.6	790.2
10	−−	9	9	755.6 ± 52.7	790.8
11	++	11	11	601.2 ± 24.4	588.4

The experimental results are the means of two replicates of 1000-fold diluted spore samples. The symbols in the model column mean each factor at five different levels (−a, −, 0, +, A). The variables at a central coded value are considered at zero

**Table 5:** Analysis of variance (ANOVA) for response surface quadratic models for spore production based on DPA florescence detection by metal ion-optimized RSM and sequential raw material-optimized RSM in submerged fermentation

Term	Metal ion-optimized RSM	Raw material-optimized RSM
*P* > *F*	< 0.0001	0.0059
R^2^	0.9400	0.9328
Adj. R^2^	0.8701	0.8656
Root mean square error	3.7142	27.281
Mean	282.3	715.0
Response surface solution	Maximum	Maximum

The value of “*P* > *F*” less than 0.05 indicates the model terms are significant

**Fig. 3:**
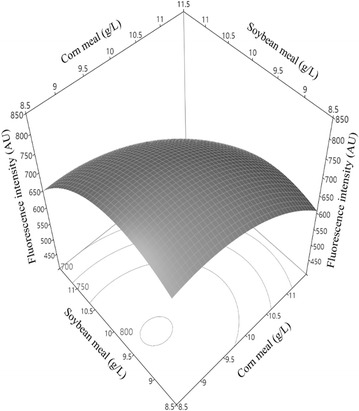
Response surface plots for spore production caused by soybean meal and corn meal

The optimization of the raw materials were also successful and greatly increased the spore yields with the value of “*P* > *F*” = 0.0059. The *R*^2^ and Adj. *R*^2^ were 0.9328 and 0.8656, respectively. The model terms *x*_6_ and $x_{6}^{2}$ were found to be significant (*P* = 0.0298 and 0.0195, respectively). The response surface plots had a downward opening convex showed the response surface maximal point was 802.03 AU (Fig. 3), which was about 2.7 times of the value in the ion-optimized RSM. The critical variable concentrations for predicted maximum spore yields were as follows: corn meal (*x*_5_) = 9.0 g/L and soybean meal (*x*_6_) = 9.5 g/L, respectively.

### Verification for spore production after optimization

The spore production results were verified to check the accuracy of the models over three replicates (Table 6). The results showed that the experimental values were very close to the predicted values, and the optimization models were validated. Moreover, the calculated colony concentrations based on the standard curves in Fig. 1 were also close to the practical measured colony concentrations (Table 6). The results indicated that the spore yield detected by fluorimetry assay were consistent to that by plate counting assay.

**Table 6:** Verification for spore production after two-step RSM optimization procedures

Optimization procedures	DPA fluorimetry assay			Plate counting assay
	Predicted fluorescence intensity (AU)	Observed fluorescence intensity (AU)	Calculated colony concentrations (CFU/mL)	Measured colony concentrations (CFU/mL)
Control	–	98.6 ± 8.9	(9.01 ± 0.03) × 10^8^	(9.10 ± 0.28) × 10^8^
Metal ion-optimized RSM	300.2	303.3 ± 22.4	(2.94 ± 0.13) × 10^9^	(3.10 ± 1.41) × 10^9^
Raw material-optimized RSM	802.0	803.3 ± 28.3	(7.94 ± 0.20) × 10^9^	(8.05 ± 0.70) × 10^9^

From the verification experiments, the optimized media (glucose 8 g/L, beef extract 7.2 g/L, corn meal 9.0 g/L, soybean meal 9.5 g/L, Mn^2+^ 1.0 mM, Fe^2+^ 3.0 mM and Ca^2+^ 2.1 mM) gave an 8.8-fold increase in the spore yield compared with the control (glucose 8 g/L, beef extract 7.2 g/L, NaCl 10 g/L). The experimental values measured by plate counting assay reached (8.05 ± 0.70) × 10^9^ CFU/mL (n = 3).

## Discussion

Several studies have been performed on the enhancement of spore production, and the top 2 highest documented spore concentrations of *Bacillus* undergoing submerged fermentation were 1.56 × 10^10^ CFU/mL (Chen et al. 2010) and 7 × 10^10^ CFU/mL (Khardziani et al. 2017), respectively. Both of these high spore yields were observed in the fermentation of *B. subtilis*. The spore yields obtained in this study are the highest levels in *B. amyloliquefaciens* fermentation compared to other reports, whose yields range from 5.93 × 10^8^ CFU/mL (Rao et al. 2007) to 3.82 × 10^9^ CFU/mL (Tzeng et al. 2008). Moreover, higher spore yield could be achieved by optimizing the culture or fermentation conditions in bioreactors with better ventilation and agitation using an optimized medium as a base (Khardziani et al. 2017).

This study focused on factors that previous reports had suggested to influence spore production (Chen et al. 2010; Khardziani et al. 2017; Kihm et al. 1988; Shi and Zhu 2007). The final result in the study showed that optimizing the type and concentration of metal ions and raw materials improved spore yields by 3.4- and 8.8-fold, respectively (Table 6). The metal ions likely played a role in activating enzyme systems necessary for sporulation (Kolodziej and Slepecky 1964). Manganese and iron are indispensable for sporulation and participate in the synthesis of *Bacillus*’s secondary metabolites, such as antibiotics and peptides (Granger et al. 2011; Greene and Slepecky 1972; Oh and Freese 1976). Calcium acts as an important component of spores by chelating with DPA (Ca-DPA) and helps to improve heat resistance (Levinson et al. 1961). This study found similar results on metal ions’ contribution to spore production (see Table 1). The single-factor experiment identified Mn^2+^, Fe^2+^ and Ca^2+^ as having a significantly positive effect on spore production. In contrast with another report (Kihm et al. 1988), the inclusion of zinc had a significantly negative effect on sporulation in the present study (*P* < 0.05). The results suggest that different strains might have different response to metal ions the in medium and using a thorough screening procedure is important before optimizing the concentration of metal ions. The inclusion of raw materials in the medium greatly improved spore yields both in the current study and other reports (Chen et al. 2010; Khardziani et al. 2017; Posada-Uribe et al. 2015). Generally, proteinase and amylase activity are similar across *Bacillus* species, and *B. amyloliquefaciens* BS-20 showed more enzyme activity than other *Bacillus* probiotics in our previous studies (data not shown). The gradually hydrolyzed substrates from protein and starch in the raw materials provides nutrients for *Bacillus* growth and spore production, which could also alleviate possible catabolite repression on sporulation caused by glucose (Chen et al. 2010; Shi and Zhu 2007).

More importantly, the current study demonstrated the use of DPA fluorimetry assays as an alternative to traditional plate counting for quantifying spore concentration in the optimization procedures. From the linear curve in Fig. 1, it can be seen that the LOD in this study (8000 spores/mL) was close to the lowest LOD (1000 spores/mL) identified in the literature (Pellegrino et al. 2002). The LOD was low enough to allow for the quantification of spore concentrations since spore yields in fermented cultures are often above 10^8^ spores/mL. Moreover, the DPA fluorimetry assay used in this study is very simple, and the fluorescent complex was produced by just mixing the diluted DPA samples, europium, and the chelating agent CyDTA. The fluorescence intensity was readily measured by a fluorescence spectrophotometer or microplate readers (Pellegrino et al. 2002). Additionally, DPA fluorimetry assay allowed fast and synchronous detection of many samples in the statistical optimization experiments. For example, in the ion-optimized RSM experiment of this study, 27 runs with 2 replicates were carried out simultaneously and all the 54 samples could be detected in 1 h by the fluorescence spectrophotometer. However, in the plate counting assay, the spore concentration of one sample was achieved by plating three tenfold dilutions of spore suspensions with at least three replicates for each dilution. Therefore, at least 3 × 3 × 54 plates were required and the colonies were finally counted after at least 24 h cultivation. Based on the results found by the DPA fluorimetry assay (presented in Table 6), it was demonstrated that the optimization techniques described in this paper provided an easy and feasible way to enhance spore production. Finally, from the optimized and verified results in this study, a DPA fluorimetry assay was successfully applied and provided a general analytical method for assessing spore concentrations with less work and time than a plate-counting assay would require.

## Abbreviations

AU: arbitrary units
CCTCC: China Center for Type Culture Collection
CCD: central composite design
CFU: colony-forming units
CyDTA: cyclohexanediamine tetraacetic acid
DPA: dipicolinic acid
LB: Luria–Bertani
LOD: limit of detection
OD: optical density
PMT: photomultiplier tube
RSM: response surface methodology
SD: standard deviation
